# SSRIs Increase Risk of Blood Transfusion in Patients Admitted for Hip Surgery

**DOI:** 10.1371/journal.pone.0095906

**Published:** 2014-05-21

**Authors:** Hermien Janneke Schutte, Sofie Jansen, Matthias U. Schafroth, J. Carel Goslings, Nathalie van der Velde, Sophia E. J. A. de Rooij

**Affiliations:** 1 Section of Geriatric Medicine, Department of Medicine, Academic Medical Center, Amsterdam, The Netherlands; 2 Department of Orthopedic Surgery, Academic Medical Center, Amsterdam, The Netherlands; 3 Trauma Unit, Department of Surgery, Academic Medical Center, Amsterdam, The Netherlands; German Red Cross Blood Service Frankfurt, Germany

## Abstract

**Background:**

Recent studies have shown that an increased bleeding tendency can be caused by Selective Serotonin Reuptake Inhibitors (SSRI) use. We aimed to investigate the occurrence and risk of blood transfusion in SSRI users compared to non-SSRI users in a cohort of patients admitted for hip-surgery.

**Methods:**

We conducted a retrospective cohort study of patients who underwent planned or emergency hip surgery from 1996 to 2011 in the Academic Medical Center in Amsterdam. Primary outcome measure was risk of blood transfusion. Secondary outcome measures were pre- and postoperative hemoglobin level. Multivariate logistic regression was used to adjust for potential confounders.

**Results:**

One-hundred and fourteen SSRI users were compared to 1773 non-SSRI users. Risk of blood transfusion during admission was increased for SSRI users in multivariate analyses (OR 1.7 [95% CI 1.1–2.5]). Also, pre-operative hemoglobin levels were lower in SSRI users (7.8±1.0 mmol/L) compared to non-SSRI users (8.0±1.0 mmol/L) (p = 0.042)), as were postoperative hemoglobin levels (6.2±1.0 mmol/L vs. 6.4±1.0 mmol/L respectively) (p = 0.017)).

**Conclusions:**

SSRI users undergoing hip surgery have an increased risk for blood transfusion during admission, potentially explained by a lower hemoglobin level before surgery. SSRI use should be considered as a potential risk indicator for increased blood loss in patients admitted for hip surgery. These results need to be confirmed in a prospective study.

## Introduction

Peri-operative blood transfusions increase the relative risk of postoperative infections and are associated with considerable morbidity and mortality [1], [2]. An important indication for blood transfusions is major peri-operative blood loss [3]. The amount of peri-operative blood loss can be negatively influenced by the use of drugs that increase bleeding tendency, such as anti-thrombotic drugs [4]. However, increased bleeding tendency is also associated with the use of Selective Serotonin Reuptake Inhibitors (SSRIs) [5]–[8]. In a recent large cohort study, peri-operative use of SSRIs was even directly associated with a higher risk of adverse events, such as in-hospital mortality and hospital readmission within 30 days [9].

In the Netherlands, the use of SSRIs is widely spread with over 500,000 prescribed patients in 2011 [10]. The most common indications for SSRI prescription in general practice are depression (53.5%) and anxiety (18.7%) [11].

Several studies have shown that use of SSRIs is associated with an increased risk of gastrointestinal bleeding. Within studies, associations varied from modest to strong [5]–[8], [12]. Despite these outcomes, only a few studies have addressed the association between SSRI use and peri-operative blood loss. These reference studies showed ambiguous result [13], [14]. Consequently, the association between SSRI use and increased risk of peri-operative bleeding remains unclear. As hip surgery is known to be the leading indication for blood transfusion in orthopedic patients [15], we aimed to study whether SSRIs increase risk of blood transfusion during admission in a cohort of patients undergoing hip surgery.

## Methods

### Setting and population

The current study was performed within a large cohort of hip surgery patients that consisted of all consecutive patients admitted for either planned or emergency hip surgery from 1996 to 2011 in an academic hospital. This cohort was gathered for a study in which the occurrence of ECG abnormalities was studied in patients undergoing hip surgery [16]. Inclusion criteria were age ≥ 50 years, pre-operative ECG present in hospital records. Exclusion criteria were previous hip fracture, hip fracture due to High Energy Trauma (HET) and pathological fracture due to malignancy or other diseases. Hip surgeries included all proximal femur fracture surgeries, including osteosynthesis and (hemi-) arthroplasty. This study was conducted according to the principles expressed in the Declaration of Helsinki. The medical ethics committee of the Academic Medical Center (Amsterdam) approved the conduction of this study and waived the necessity to obtain informed consent from participants because of the observational design.

### Covariate information

The following covariates were collected: age at the time of surgery (years), gender, Charlson Comorbidity Index (CCI) [17], home situation at time of admission, type of surgery, date of surgery, estimated glomerular filtration rate (eGFR) by the Modification of Diet in Renal Decease (MDRD) equation [18], previously diagnosed clinical depression and all drug use. Home situation was defined as community dwelling (living at home or in assisted living) or living in an institution (nursing home, home for elderly or other hospital). Type of surgery was defined as either emergency hip surgery (all proximal femur fractures with subsequent surgery) or planned hip surgery (treatment for osteoarthritis or avascular necrosis through total hip replacement). Date of surgery was categorized as before 2004 or from 2004 and onwards, corresponding the change in blood transfusion guidelines. All data were retrieved from patients' medical records.

### Markers for increased bleeding tendency

Primary outcome measure was the occurrence of blood transfusion during hospital stay (pre-, peri- and postoperative). In 1998, local guidelines advised pre-operative blood transfusion from a hemoglobin level of 4.5–5.0 mmol/L and a postoperative blood transfusion from 4.5–5.5 mmol/L. In 2004, new national guidelines for blood transfusion introduced the prevailing ‘4-5-6 rule’, which combined age, American Society of Anesthesiologists (ASA) score and hemoglobin level [19], [20].

Secondary outcome variables were the amount of packed cells administered and pre- and postoperative serum hemoglobin levels (mmol/L). To investigate peri-operative loss of hemoblobin, a delta-hemoglobin level was defined (pre-operative hemoglobin level subtracted from the postoperative hemoglobin level). Pre-operative serum hemoglobin was defined as most recent hemoglobin level before surgery within 72 hours. Postoperative hemoglobin level was obtained from the first postoperative blood samples within 72 hours.

### SSRI and other drug use

Medication use at time of admission was verified through hospital charts, pharmacy notes and/or referral letters. Drugs were listed and grouped according to ATC codes. SSRI use included all types of SSRIs registered and prescribed at the time of surgery. All anti-thrombotic agents, (heparin, vitamin K antagonists and platelet aggregation inhibitors), were also registered. Furthermore, non-steroidal anti-inflammatory drugs (NSAIDs) and anti-rheumatic drugs were registered.

### Health care consumption

Other variables that were possibly related to increased bleeding tendency in the setting of major surgery were explored. Duration of surgery was defined as the actual duration of surgery, excluding duration of pre- and postoperative care in the operating room and recovery. Duration of hospital admission (days) and hospital readmission within 30 days after admission (all causes included) were also gathered.

### Statistical analysis

Baseline characteristics and outcome variables were calculated either as the mean and SD or as frequency and percentage of occurrence. Independent samples T-tests were used for continuous variables and Chi-square test for dichotomous variables. Histograms of continuous variables were analyzed to affirm normal distribution. In non-normally distributed data, we used Mann-Whitney U and Kruskal-Wallis non-parametric tests. A p-value ≤0.05 was considered statistically significant. To assess for potential confounders, multivariate logistic regression analyses were performed. Variables were considered confounders when they altered the coefficient of the main variable by more than ten percent [21]. In the multivariate logistic regression analyses, multiple imputation was used for variables that had more than ten percent missing values [22]. As it is known that patients undergoing emergency hip-surgery are usually frailer than patients undergoing planned hip-surgery, potentially influencing our results, we repeated our analyses in patients undergoing emergency hip-surgery exclusively.

Finally, potential influence by change in guidelines was analyzed by testing for potential interaction (interaction term between date of blood transfusion categorized as before 2004, or from 2004 and onwards and SSRI use).

Statistical analyses were performed with SPSS (IBM SPSS Statistics version 19.0).

## Results

In total, 1887 hip surgery patients were included, of whom 114 were SSRI users. Table 1 represents the baseline characteristics of SSRI users and non-users. Age and gender were equally distributed between both groups. On average, SSRI users had more comorbidity, more often underwent emergency hip surgery and more often lived in institutions compared to non-SSRI users. Date of surgery, estimated GFR and use of anti-thrombotic and non-steroidal anti-inflammatory drugs (NSAIDS) were equal in both groups.

**Table 1:** Baseline Characteristics of SSRI users and non-SSRI users.

	SSRI users	non- SSRI users	
	(n = 114)	(n = 1773)	
Variable	Mean (±)/n (%)	Mean (±)/n (%)	p
Age at the time of surgery (years)	78.1 (±11.7)	77.0 (±11.4)	0.317
Gender			
*Female*	85 (74.6%)	1269 (71.0%)	0.492
Type of surgery			
*Acute*	99 (86.8%)	1213 (68.4%)	<0.001
Date of surgery			
*In 2004 and onwards*	55 (48.2%)	755 (42.6%)	0.236
Home situation at admission (n = 1739)			
*Communitiy dwelling*	55 (48.2%)	1326 (74.8%)	<0.001
Charlson Comorbidity Index	1.5 (±1.5)	1.2 (±1.5)	0,029
eGFR (n = 1822)	85.5 (±34.1)	83.1 (±30.0)	0,463
Clinical Depression	24 (32.0%)	90 (5.0%)	<0.001
Anti-thrombotic drug use	41 (36.0%)	595 (33.6%)	0.598
NSAIDs/anti- rheumatic drug use	15 (13.2%)	271 (15.3%)	0.539

SSRI =  Selective Serotonin Reuptake Inhibitor;

eGFR =  estimated Glomerular Filtration Rate;

NSAIDs =  Non-Steroidal Anti Inflammatory Drugs;

± =  standard deviation.

Table 2 represents the association of SSRI use with different markers for increased bleeding tendency. Forty-eight percent of SSRI users underwent blood transfusion during admission, compared to 38% of non-SSRI users (p = 0.021). Pre-operative hemoglobin levels were lower in SSRI users than non-SSRI users (p = 0.042), as were postoperative hemoglobin levels (p = 0.017). Delta-hemoglobin level showed no difference between SSRI users and non-users. Length of hospital stay, duration of surgery and readmission rate within thirty days were equal in both groups.

**Table 2:** Markers for increased bleeding tendency and health care consumption.

	SSRI users	non-SSRI users	
	(n = 114)	(n = 1773)	
	Mean (±)/n (%)	Mean (±)/n (%)	p
Blood transfusion during admission	55 (48.2%)	663 (37.7%)	0.021
Amount of packed cells administered during admission (n = 718) †	2.9 (±1.9)	3.1 (±3.1)	0.631
Pre-operative hemoglobin level (n = 1499) (mmol/L) ‡	7.8 (±1.0)	8.0 (±1.0)	0.042
Postoperative hemoglobin level (n = 1743) (mmol/L) ‡	6.2 (±1.0)	6.4 (±1.0)	0.017
Delta hemoglobin level (n = 1323) (mmol/L) §	1.7 (±0.8)	1.7 (±0.9)	0.624
Readmission within 30 days	9 (7.9%)	92 (5.2%)	0.213
Duration of surgery (n = 1884) (minutes)	91.0 (43.4%)	93.9 (38.7%)	0.449
Length of hospital stay (days)	13.9 (±11.8)	15.5 (±15.5)	0.288

† =  only transfused patients included;

‡ =  pre-operative hemoglobin level: <72 hours before surgery, postoperative hemoglobin level: <72 hours after surgery;

§ =  (postoperative hemoglobin - pre-operative hemoglobin);

SSRI =  Selective Serotonin Reuptake Inhibitor;

± =  standard deviation.

Table 3 shows associations between blood transfusion during hospitalization and use of SSRIs, anti-thrombotic drugs and NSAIDs. Model 1 represents the odds ratios adjusted for age and gender and model 2 the odds ratios adjusted for age, gender, established depressive disorder and the estimated glomerular filtration rate (variables that changed the regression coefficient by more than 10%). After adjustment in the second model, SSRI users had an increased risk of blood transfusion (OR 1.7 [95% CI 1.1–2.5]). Odds ratios for blood transfusion were also increased for anti-thrombotic drug use (OR 1.3 [1.1–1.6]) and NSAIDs/anti-rheumatic drug use (OR 1.4 [1.0–1.8]). After further adjustment for pre-operative hemoglobin levels (model 3), odds ratios for blood transfusion for all three drug types remained increased. However, the association between SSRI use and blood transfusion lost statistical significance.

**Table 3:** Odds Ratios (OR) for blood transfusion during admission (n = 1887).

	Model 1		Model 2		Model 3	
	OR	95% CI	OR	95% CI	OR	95% CI
SSRI	1.5	(1.0–2.2)	1.7	(1.1–2.5)	1.4	(0.9–2.2)
Anti-thrombotic	1.4	(1.2–1.7)	1.3	(1.1–1.6)	1.3	(1.0–1.6)
NSAIDs/anti-rheumatic drugs	1.4	(1.0–1.8)	1.4	(1.0–1.8)	1.3	(1.0–1.8)

Model 1 =  adjusted for age and gender;

Model 2 =  adjusted for age, gender, eGFR, depression;

Model 3 =  adjusted for age, gender, eGFR, depression, pre-operative hemoglobin level (<72 hours after surgery), Missing variables for pre-operative hemoglobin level and eGFR were imputed through multiple imputation;

CI =  Confidence Interval;

OR =  Odds Ratio;

aOR =  adjusted Odds Ratio;

SSRI =  Selective Serotonin Reuptake Inhibitor;

eGFR =  estimated Glomerular Filtration Rate;

NSAIDs =  Non-steroidal Anti Inflammatory Drugs.

Repeating the multivariate logistic regression in emergency hip-surgery patients exclusively (n = 1312), SSRI use remained positively associated with an increased risk of blood transfusion (OR 1.7 [95% CI 1.1–2.6]).

Finally, the interaction term between date of blood transfusion (categorized as before 2004, or from 2004 and onwards) and SSRI use in the regression models was not significant (p = 0.278).

Because of >10% missing values, for the latter analyses imputation of pre-operative hemoglobin levels and estimated glomerular filtration rate was performed.

## Discussion

The results of our study show that patients who used SSRIs at the time of hip surgery had an increased risk of blood transfusion during hospitalization. A lower pre-operative hemoglobin level in SSRI users potentially causes this greater risk.

The results of our study correspond to a study by Movig et al. In this study, risk of blood transfusion was quadrupled among SSRI users [14]. Their study participants were younger patients undergoing planned spine, knee and hip surgery, whereas our study exclusively included older patients undergoing hip surgery. Hip surgery is known to be the leading indication for blood transfusion in orthopedic patients [15]. In our cohort this may have caused more events in both SSRI users and non-SSRI users, possibly overshadowing the effects of SSRIs on bleeding tendency alone. Another study by van Haelst et al. reported an increase in peri-operative blood loss in SSRI users compared to non-SSRI users in a cohort of patients undergoing elective hip surgery [13]. However, this did not result in a significant increase in blood transfusions in SSRI users. Van Haelst et al. exclusively included elective hip surgery patients who differ from patients undergoing emergency hip surgery regarding comorbidity and drug use, which might explain the differences with our findings. The differences between our results and the mentioned studies could also be explained by the fact that hospitals employ different triggers or criteria for blood transfusion [23].

The results of our study also show that SSRI users had lower pre- and postoperative hemoglobin levels compared to non-SSRI users. As delta-hemoglobin level was equal in both groups, the lower post-operative hemoglobin level in SSRI users is most likely explained by the lower pre-operative hb levels in SSRI users. These results are comparable to both reference studies, in which lower pre-operative hemoglobin levels were also found in SSRI users, however not significant [13], [14]. Since it is unclear whether reference studies used hemoglobin levels measured within 72 hours peri-operatively, it is difficult to compare these results with ours.

Adjustment for pre-operative hemoglobin level altered the ORs for blood transfusion according to SSRI use, confirming the influence of pre-operative hemoglobin level on risk of blood transfusion. We considered pre-operative hemoglobin as an intermediate, since extra pre-operative blood loss would be a trigger for blood transfusion.

In this study's frail population, reasons for lower starting hemoglobin levels could be manifold. Our theory is that SSRI users have lower starting hemoglobin level due to increased bleeding in the hip-fracture, caused by a lower anti-platelet aggregation function. Although SSRI users more often underwent emergency hip-surgery, were more often institutionalized and had more comorbidity, the adjustment for these potential confounders did not influence our results. Also, repeating our analysis in emergency hip-surgery patients exclusively did not lead to different results.

The underlying mechanism of the increased bleeding tendency is currently attributed to the known ability of SSRIs to block the 5-HTT transporter in neurons. 5-HTT transporters in neurons have the same structure as those in platelets and can therefore cause a low serotonin level in platelets, which has a decreasing effect on the platelet aggregation function [24]. This theory is currently accepted; however recently a new theory suggests that SSRIs cause additional damage to the gastrointestinal mucosa, which might lead to gastrointestinal bleeding [25]. Potentially, SSRI users had more (occult) gastro-intestinal blood loss. In this retrospective study however, potential mucosal defects and gastro-intestinal blood loss could not be objectivized and therefore we can only speculate on this potential pathway.

Literature also suggests that a clinical depression is associated with increased or decreased platelet aggregation [26], [27]. However, not all study participants who used SSRIs had a diagnosis of clinical depression. In our analysis, we therefore regarded clinical depression as a potential confounder. Correcting for depression in our analyses did however not change the significance of our results, indicating that SSRIs were not acting just as a mediator for clinical depression.

Our study has several strengths. First of all, the size of this study cohort is considerably larger than two other studies. Furthermore, considering the mean age, comorbidity and type of surgery of the study subjects, our cohort probably represents the population that is mostly troubled by adverse effects of SSRI use [28].

However, due to the retrospective design of the study, interpretation of our findings is to some extent limited. The registration of SSRIs relied on the registrations in surgical charts. Detailed information on medication use (such as dose and duration) was inconsistent due to the routine registration diversity among physicians. SSRI use was therefore potentially underreported, which might have led to a dilution of the actual effect size. Furthermore, the indication for blood transfusion in hospitals is currently customarily based on the ASA score and hemoglobin level, therefore it does not depend solely on the amount of blood loss [3]. As we have no reason to assume that this was differential for both groups, we do not consider confounding by indication.

The hospital's criteria for blood transfusion changed during the study period [3]. However, analysis showed no interaction between date of blood transfusion (categorized as before 2004, or from 2004 and onwards) and SSR use. Therefore, change in guidelines was not considered as a moderator.

Finally, it is possible that the prescription of certain anti-coagulant drugs was stopped a few days before surgery. Therefore the association between use of these drugs and blood transfusion could be underestimated. However, it is unlikely that this has affected our main results. But to fully compare SSRIs with these other drugs, more information regarding actual drug use would be needed.

This study showed that SSRI users have an increased risk of blood transfusion during hospitalization compared to non-SSRI users (OR1.7 [95%CI 1.1–2.5]). A lower pre-operative hemoglobin level in SSRI users most likely caused this increased risk of blood transfusion. The increased risk has implications for clinical practice, especially for the peri-operative care of frail older patients who are at increased risk of peri-operative complications per sé. Use of SSRIs around surgery is momentarily not mentioned as a potential risk indicator for increased bleeding tendency in orthopedic and anesthetic guidelines. However, our findings need to be validated in a prospective cohort study.
